# Tri­carbonyl­chlorido­(6′,7′-di­hydro-5′*H*-spiro­[cyclo­pentane-1,6′-dipyrido[3,2-*d*:2′,3′-*f*][1,3]diazepine]-κ^2^ *N* ^1^,*N* ^11^)rhenium(I)

**DOI:** 10.1107/S1600536813023076

**Published:** 2013-09-04

**Authors:** Oliver R. Clegg, Lindsay P. Harding, John W. Miller, Craig R. Rice

**Affiliations:** a Department of Chemical & Biological Sciences, University of Huddersfield, Queensgate, Huddersfield HD1 3DH, England

## Abstract

In the title compound, [ReCl(C_15_H_16_N_4_)(CO)_3_], the Re^I^ ion is coordinated in a distorted octa­hedral geometry by one Cl atom, two N atoms of the bidentate ligand and three carbonyl groups. The cyclo­pentane group is orientated in a *transoid* fashion with respect to the chloride ligand. The dihedral angle between the pryridine rings is 10.91 (12)°. In the crystal, N—H⋯Cl hydrogen bonds link complex mol­ecules, forming a two-dimensional network parallel to (001).

## Related literature

For a review of the photophysical properties of Re–polypyridyl complexes, see: Coleman *et al.* (2008 ▶). For the synthesis of [Re(3,3′-di­amino-2,2′-bi­pyridine)(CO)_3_Cl] and for the preparation of oxo-steroid derivatives of [Re(3,3′-di­amino-2,2′-bi­pyridine)(CO)_3_Cl], see: Bullock *et al.* (2012 ▶). For the reaction of [Re(3,3′-di­amino-2,2′-bi­pyridine)(CO)_3_Cl] with ketones, see: Clayton *et al.* (2008 ▶). For the structure of the cyclo­hexane analog of the title compound, see: Clegg *et al.* (2013 ▶).
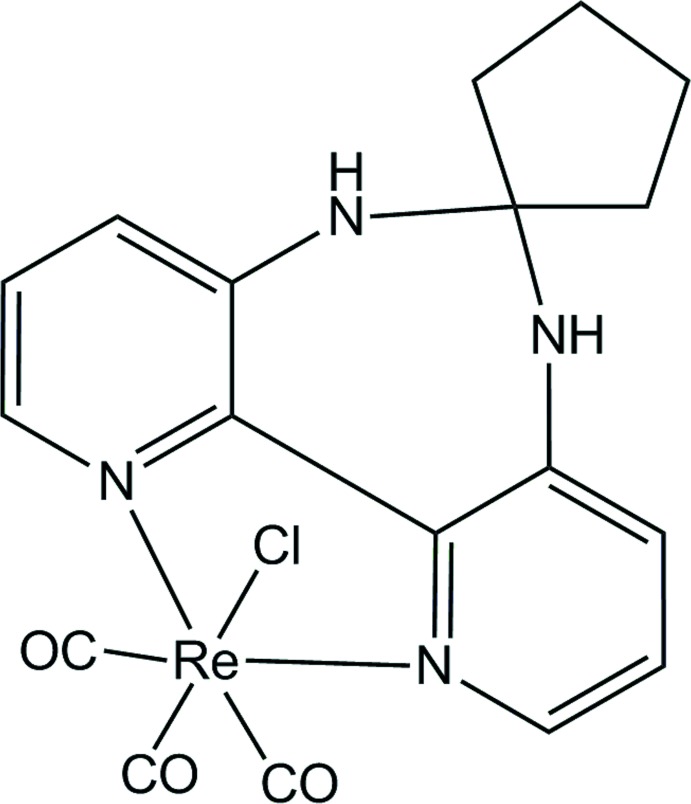


## Experimental

### 

#### Crystal data

[ReCl(C_15_H_16_N_4_)(CO)_3_]
*M* *_r_* = 558.00
Orthorhombic, 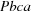

*a* = 12.1162 (5) Å
*b* = 11.9638 (5) Å
*c* = 24.9181 (9) Å
*V* = 3612.0 (2) Å^3^
*Z* = 8
Mo *K*α radiation
μ = 6.90 mm^−1^
*T* = 150 K
0.50 × 0.50 × 0.20 mm

#### Data collection

Bruker APEXII CCD diffractometer
Absorption correction: multi-scan (*SADABS*; Bruker, 2009 ▶) *T*_min_ = 0.16, *T*_max_ = 0.34
45382 measured reflections
10757 independent reflections
7941 reflections with *I* > 2σ(*I*)
*R*_int_ = 0.053

#### Refinement

*R*[*F* ^2^ > 2σ(*F* ^2^)] = 0.037
*wR*(*F* ^2^) = 0.075
*S* = 1.04
10757 reflections
244 parameters
H-atom parameters constrained
Δρ_max_ = 2.28 e Å^−3^
Δρ_min_ = −5.29 e Å^−3^

### 

Data collection: *APEX2* (Bruker, 2009 ▶); cell refinement: *SAINT* (Bruker, 2009 ▶); data reduction: *SAINT*; program(s) used to solve structure: *SHELXS97* (Sheldrick, 2008 ▶); program(s) used to refine structure: *SHELXL97* (Sheldrick, 2008 ▶); molecular graphics: *OLEX2* (Dolomanov *et al.*, 2009 ▶); software used to prepare material for publication: *OLEX2*.

## Supplementary Material

Crystal structure: contains datablock(s) global, I. DOI: 10.1107/S1600536813023076/lh5644sup1.cif

Structure factors: contains datablock(s) I. DOI: 10.1107/S1600536813023076/lh5644Isup2.hkl

Additional supplementary materials:  crystallographic information; 3D view; checkCIF report

## Figures and Tables

**Table 1:** Hydrogen-bond geometry (Å, °)

*D*—H⋯*A*	*D*—H	H⋯*A*	*D*⋯*A*	*D*—H⋯*A*
N3—H3*A*⋯Cl1^i^	0.88	2.53	3.363 (3)	158
N4—H4⋯Cl1^ii^	0.88	2.65	3.419 (2)	147

Symmetry codes: (i) 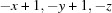; (ii) 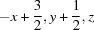.

**Fig. 1.**
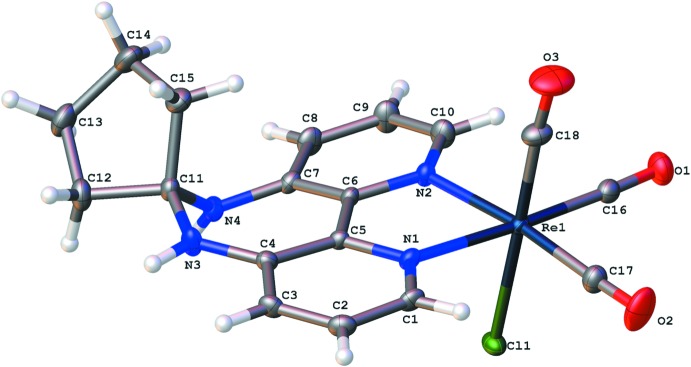
The molecular structure of the title compound with displacement ellipsoids shown for non-H atoms at the 50% probability level.

## supplementary crystallographic information

### 1. Comment

The title complex was prepared as part of a larger study into conjugation of [Re(3,3'-diamino-2,2'-bipyridine)(CO)_3_Cl] with oxo-steroids to form luminescent derivatives (Bullock *et al.* 2012). These steroids contain a cyclopentyl ring (ring D) with a ketone group in the 17-position; therefore, cyclopentanone was used as a model compound to examine the potential reactivity of such steroids with the rhenium complex. The photophysical properties of Re-polypyridyl complexes have been studied (Coleman *et al.*, 2008) as well as the reaction of [Re(3,3'-diamino-2,2'-bipyridine)(CO)_3_Cl] with ketones (Clayton *et al.*, 2008).

Single-crystal X-ray analysis of the product gave the structure shown in Fig. 1. The rhenium centre adopts a distorted octahedral coordination geometry and is coordinated by two nitrogen atoms from 3,3'-diamino-2,2'-bipyridyl and two carbonyl ligands in the equatorial positions (Re—N distances 2.158 (2) - 2.169 (2) Å, Re—C distances 1.924 (3) - 1.925 (3) Å). Carbonyl and chloride ligands occupy the axial positions (Re—C distance 1.891 (3) Å, Re—Cl distance 2.5046 (6) Å). The cyclopentyl ring is orientated in a *trans*-oid fashion with respect to the chloride ligand on the rhenium centre. In the crystal, N—H···O hydrogen bonds (see, Table 1) link complex molecules to form a two-dimensional network parallel (001).

A similar compound has been prepared using cyclohexanone instead of cyclopentanone. This compound is essentially isostructural with the compound reported here (Clegg *et al.* 2013).

### 2. Experimental

To a solution of [Re(3,3'-diamino-2,2'-bipyridine)(CO)_3_Cl] in dichloromethane was added cyclopentanone (10 µ*L*, *ca* 2 eq.) and a few grains of camphorsulfonic acid. The solution was stirred at room temperature for 2 h. The resulting precipitate was filtered *in vacuo*, washed with dichloromethane and dried, affording the product as a yellow solid. Slow evaporation of an acetonitrile solution of the complex gave yellow crystals suitable for X-ray analysis.

### 3. Refinement

All non-hydrogen atoms were refined anisotropically. Hydrogen atoms on *sp*^2^ and *sp*^3^ carbons were placed in calculated positions (C—H = 0.95 - 0.99Å) and refined with riding constraints and with isotropic displacement parameters 1.2 *x* their parent carbon atoms. H atoms on the nitrogen atoms were treated similarly with N—H = 0.88Å.

### Crystal data

**Table:**

[ReCl(C_15_H_16_N_4_)(CO)_3_]	*D*_x_ = 2.052 Mg m^−^^3^
*M**_r_* = 558.00	Mo *K*α radiation, λ = 0.71073 Å
Orthorhombic, *P**b**c**a*	Cell parameters from 9921 reflections
*a* = 12.1162 (5) Å	θ = 2.9–39.3°
*b* = 11.9638 (5) Å	µ = 6.90 mm^−^^1^
*c* = 24.9181 (9) Å	*T* = 150 K
*V* = 3612.0 (2) Å^3^	Block, yellow
*Z* = 8	0.50 × 0.50 × 0.20 mm
*F*(000) = 2144	

### Data collection

**Table:**

Bruker APEXII CCD diffractometer	10757 independent reflections
Graphite monochromator	7941 reflections with *I* > 2σ(*I*)
Detector resolution: 8.3333 pixels mm^-1^	*R*_int_ = 0.053
φ and ω scans	θ_max_ = 39.4°, θ_min_ = 2.3°
Absorption correction: multi-scan (*SADABS*; Bruker, 2009)	*h* = −21→20
*T*_min_ = 0.16, *T*_max_ = 0.34	*k* = −21→13
45382 measured reflections	*l* = −44→41

### Refinement

**Table:**

Refinement on *F*^2^	Primary atom site location: structure-invariant direct methods
Least-squares matrix: full	Secondary atom site location: difference Fourier map
*R*[*F*^2^ > 2σ(*F*^2^)] = 0.037	Hydrogen site location: inferred from neighbouring sites
*wR*(*F*^2^) = 0.075	H-atom parameters constrained
*S* = 1.04	*w* = 1/[σ^2^(*F*_o_^2^) + (0.0093*P*)^2^ + 8.4209*P*] where *P* = (*F*_o_^2^ + 2*F*_c_^2^)/3
10757 reflections	(Δ/σ)_max_ = 0.002
244 parameters	Δρ_max_ = 2.28 e Å^−^^3^
0 restraints	Δρ_min_ = −5.29 e Å^−^^3^

### Special details

**Table:**

Geometry. All e.s.d.'s (except the e.s.d. in the dihedral angle between two l.s. planes) are estimated using the full covariance matrix. The cell e.s.d.'s are taken into account individually in the estimation of e.s.d.'s in distances, angles and torsion angles; correlations between e.s.d.'s in cell parameters are only used when they are defined by crystal symmetry. An approximate (isotropic) treatment of cell e.s.d.'s is used for estimating e.s.d.'s involving l.s. planes.
Refinement. Refinement of *F*^2^ against ALL reflections. The weighted *R*-factor *wR* and goodness of fit *S* are based on *F*^2^, conventional *R*-factors *R* are based on *F*, with *F* set to zero for negative *F*^2^. The threshold expression of *F*^2^ > σ(*F*^2^) is used only for calculating *R*-factors(gt) *etc*. and is not relevant to the choice of reflections for refinement. *R*-factors based on *F*^2^ are statistically about twice as large as those based on *F*, and *R*- factors based on ALL data will be even larger.

### Fractional atomic coordinates and isotropic or equivalent isotropic displacement parameters (Å^2^)

**Table:**

	*x*	*y*	*z*	*U*_iso_*/*U*_eq_	
Re1	0.454601 (7)	0.268496 (9)	0.14683 (4)	0.01301 (3)	
Cl1	0.61326 (5)	0.24039 (6)	0.08383 (3)	0.01986 (12)	
N1	0.39870 (16)	0.38723 (19)	0.08695 (8)	0.0143 (4)	
N2	0.53943 (17)	0.4231 (2)	0.16399 (9)	0.0156 (4)	
N3	0.4418 (2)	0.6806 (2)	0.04288 (10)	0.0216 (5)	
H3A	0.4175	0.7169	0.0145	0.026*	
N4	0.61142 (17)	0.6801 (2)	0.09197 (9)	0.0170 (4)	
H4	0.6735	0.6957	0.0752	0.02*	
O1	0.55829 (19)	0.1241 (2)	0.23572 (10)	0.0301 (5)	
O2	0.3300 (2)	0.0584 (2)	0.10992 (12)	0.0425 (7)	
O3	0.2621 (2)	0.3006 (3)	0.22352 (12)	0.0467 (7)	
C1	0.3219 (2)	0.3577 (2)	0.05113 (10)	0.0177 (5)	
H1	0.2898	0.2854	0.0533	0.021*	
C2	0.2877 (2)	0.4305 (3)	0.01061 (10)	0.0191 (5)	
H2	0.2351	0.4073	−0.0154	0.023*	
C3	0.3314 (2)	0.5352 (3)	0.00911 (10)	0.0182 (5)	
H3	0.3084	0.5854	−0.0182	0.022*	
C4	0.41063 (19)	0.5711 (2)	0.04738 (9)	0.0149 (4)	
C5	0.44682 (18)	0.4917 (2)	0.08588 (9)	0.0131 (4)	
C6	0.53279 (19)	0.5069 (2)	0.12741 (9)	0.0133 (4)	
C7	0.60801 (19)	0.5965 (2)	0.13002 (10)	0.0153 (4)	
C8	0.6856 (2)	0.5981 (3)	0.17185 (12)	0.0211 (5)	
H8	0.737	0.6578	0.1742	0.025*	
C9	0.6876 (2)	0.5138 (3)	0.20945 (12)	0.0242 (6)	
H9	0.7393	0.515	0.2381	0.029*	
C10	0.6127 (2)	0.4277 (3)	0.20445 (11)	0.0219 (5)	
H10	0.6129	0.3697	0.2305	0.026*	
C11	0.5101 (2)	0.7430 (2)	0.07960 (11)	0.0173 (5)	
C12	0.5445 (3)	0.8552 (3)	0.05556 (13)	0.0249 (6)	
H12A	0.481	0.8927	0.0382	0.03*	
H12B	0.604	0.8453	0.0287	0.03*	
C13	0.5857 (3)	0.9228 (3)	0.10410 (14)	0.0303 (7)	
H13A	0.5668	1.0029	0.1	0.036*	
H13B	0.6667	0.9157	0.108	0.036*	
C14	0.5256 (3)	0.8717 (3)	0.15337 (13)	0.0319 (7)	
H14A	0.5796	0.84	0.179	0.038*	
H14B	0.4815	0.9295	0.1721	0.038*	
C15	0.4503 (2)	0.7797 (3)	0.13103 (13)	0.0228 (5)	
H15A	0.3758	0.8091	0.1229	0.027*	
H15B	0.4435	0.7168	0.1567	0.027*	
C16	0.5196 (2)	0.1764 (3)	0.20172 (11)	0.0189 (5)	
C17	0.3787 (2)	0.1363 (3)	0.12295 (12)	0.0228 (5)	
C18	0.3353 (2)	0.2923 (3)	0.19437 (12)	0.0238 (6)	

### Atomic displacement parameters (Å^2^)

**Table:**

	*U*^11^	*U*^22^	*U*^33^	*U*^12^	*U*^13^	*U*^23^
Re1	0.01383 (4)	0.01096 (5)	0.01425 (4)	0.00013 (3)	0.00075 (3)	−0.00102 (3)
Cl1	0.0199 (2)	0.0217 (3)	0.0180 (2)	0.0056 (2)	0.00425 (19)	0.0036 (2)
N1	0.0139 (7)	0.0123 (10)	0.0166 (8)	0.0004 (7)	−0.0027 (6)	−0.0025 (7)
N2	0.0174 (8)	0.0125 (10)	0.0170 (8)	0.0000 (7)	−0.0035 (7)	0.0010 (7)
N3	0.0285 (11)	0.0178 (13)	0.0183 (9)	−0.0037 (9)	−0.0082 (8)	0.0047 (8)
N4	0.0163 (8)	0.0146 (11)	0.0202 (9)	−0.0006 (7)	0.0012 (7)	0.0025 (8)
O1	0.0363 (12)	0.0265 (13)	0.0275 (11)	0.0024 (9)	−0.0035 (9)	0.0085 (10)
O2	0.0472 (15)	0.0211 (14)	0.0590 (18)	−0.0087 (11)	−0.0202 (13)	−0.0053 (12)
O3	0.0349 (13)	0.058 (2)	0.0469 (16)	0.0043 (13)	0.0198 (12)	−0.0117 (15)
C1	0.0174 (9)	0.0164 (13)	0.0193 (10)	−0.0012 (8)	−0.0041 (8)	−0.0063 (9)
C2	0.0177 (10)	0.0242 (15)	0.0156 (10)	0.0013 (9)	−0.0036 (8)	−0.0039 (9)
C3	0.0194 (10)	0.0227 (15)	0.0126 (9)	−0.0005 (9)	−0.0032 (8)	0.0007 (9)
C4	0.0163 (9)	0.0160 (13)	0.0125 (9)	−0.0004 (8)	−0.0002 (7)	−0.0004 (8)
C5	0.0143 (8)	0.0128 (11)	0.0121 (8)	0.0008 (7)	−0.0019 (7)	−0.0009 (7)
C6	0.0166 (9)	0.0100 (11)	0.0132 (9)	−0.0001 (7)	−0.0015 (7)	−0.0010 (7)
C7	0.0157 (9)	0.0118 (12)	0.0183 (10)	0.0010 (8)	−0.0021 (8)	−0.0019 (8)
C8	0.0201 (10)	0.0182 (14)	0.0250 (12)	−0.0054 (9)	−0.0067 (9)	−0.0023 (10)
C9	0.0241 (12)	0.0233 (16)	0.0252 (13)	−0.0047 (10)	−0.0120 (10)	0.0025 (11)
C10	0.0249 (12)	0.0205 (15)	0.0205 (11)	−0.0004 (10)	−0.0085 (9)	0.0022 (10)
C11	0.0217 (10)	0.0126 (13)	0.0176 (10)	0.0001 (9)	−0.0019 (8)	0.0020 (8)
C12	0.0297 (13)	0.0180 (15)	0.0271 (13)	−0.0026 (11)	−0.0042 (11)	0.0073 (11)
C13	0.0381 (17)	0.0156 (15)	0.0373 (17)	−0.0025 (12)	−0.0048 (13)	−0.0031 (13)
C14	0.0504 (19)	0.0193 (16)	0.0259 (15)	0.0032 (14)	−0.0048 (13)	−0.0067 (12)
C15	0.0252 (12)	0.0195 (16)	0.0237 (12)	0.0053 (10)	0.0018 (9)	−0.0016 (10)
C16	0.0207 (10)	0.0160 (14)	0.0200 (11)	−0.0019 (9)	0.0019 (8)	−0.0006 (9)
C17	0.0267 (12)	0.0186 (15)	0.0232 (12)	−0.0020 (10)	−0.0037 (10)	0.0000 (10)
C18	0.0208 (11)	0.0261 (17)	0.0245 (12)	0.0018 (10)	0.0032 (9)	−0.0062 (11)

### Geometric parameters (Å, º)

**Table:**

Re1—C18	1.891 (3)	C3—H3	0.95
Re1—C17	1.924 (3)	C4—C5	1.419 (4)
Re1—C16	1.925 (3)	C5—C6	1.479 (3)
Re1—N2	2.158 (2)	C6—C7	1.409 (4)
Re1—N1	2.169 (2)	C7—C8	1.404 (4)
Re1—Cl1	2.5046 (6)	C8—C9	1.377 (4)
N1—C1	1.337 (3)	C8—H8	0.95
N1—C5	1.380 (3)	C9—C10	1.378 (4)
N2—C10	1.345 (3)	C9—H9	0.95
N2—C6	1.358 (3)	C10—H10	0.95
N3—C4	1.368 (4)	C11—C12	1.528 (4)
N3—C11	1.442 (4)	C11—C15	1.536 (4)
N3—H3A	0.88	C12—C13	1.538 (5)
N4—C7	1.379 (4)	C12—H12A	0.99
N4—C11	1.473 (3)	C12—H12B	0.99
N4—H4	0.88	C13—C14	1.553 (5)
O1—C16	1.153 (4)	C13—H13A	0.99
O2—C17	1.149 (4)	C13—H13B	0.99
O3—C18	1.150 (4)	C14—C15	1.535 (5)
C1—C2	1.396 (4)	C14—H14A	0.99
C1—H1	0.95	C14—H14B	0.99
C2—C3	1.361 (4)	C15—H15A	0.99
C2—H2	0.95	C15—H15B	0.99
C3—C4	1.420 (3)		
			
C18—Re1—C17	87.23 (13)	N4—C7—C8	118.7 (2)
C18—Re1—C16	87.36 (13)	N4—C7—C6	122.7 (2)
C17—Re1—C16	86.85 (12)	C8—C7—C6	118.6 (2)
C18—Re1—N2	96.39 (12)	C9—C8—C7	120.5 (3)
C17—Re1—N2	173.32 (11)	C9—C8—H8	119.8
C16—Re1—N2	98.89 (10)	C7—C8—H8	119.8
C18—Re1—N1	95.38 (11)	C8—C9—C10	118.3 (2)
C17—Re1—N1	100.15 (11)	C8—C9—H9	120.8
C16—Re1—N1	172.58 (10)	C10—C9—H9	120.8
N2—Re1—N1	73.97 (8)	N2—C10—C9	122.2 (3)
C18—Re1—Cl1	179.06 (11)	N2—C10—H10	118.9
C17—Re1—Cl1	93.60 (9)	C9—C10—H10	118.9
C16—Re1—Cl1	93.12 (8)	N3—C11—N4	110.3 (2)
N2—Re1—Cl1	82.74 (6)	N3—C11—C12	111.3 (2)
N1—Re1—Cl1	84.05 (6)	N4—C11—C12	107.7 (2)
C1—N1—C5	121.4 (2)	N3—C11—C15	114.0 (2)
C1—N1—Re1	120.25 (19)	N4—C11—C15	111.4 (2)
C5—N1—Re1	118.36 (15)	C12—C11—C15	101.8 (2)
C10—N2—C6	120.8 (2)	C11—C12—C13	104.0 (2)
C10—N2—Re1	119.9 (2)	C11—C12—H12A	111.0
C6—N2—Re1	118.13 (16)	C13—C12—H12A	111.0
C4—N3—C11	127.0 (2)	C11—C12—H12B	111.0
C4—N3—H3A	116.5	C13—C12—H12B	111.0
C11—N3—H3A	116.5	H12A—C12—H12B	109.0
C7—N4—C11	119.3 (2)	C12—C13—C14	105.2 (3)
C7—N4—H4	120.3	C12—C13—H13A	110.7
C11—N4—H4	120.3	C14—C13—H13A	110.7
N1—C1—C2	121.6 (3)	C12—C13—H13B	110.7
N1—C1—H1	119.2	C14—C13—H13B	110.7
C2—C1—H1	119.2	H13A—C13—H13B	108.8
C3—C2—C1	118.6 (2)	C15—C14—C13	105.9 (3)
C3—C2—H2	120.7	C15—C14—H14A	110.5
C1—C2—H2	120.7	C13—C14—H14A	110.5
C2—C3—C4	121.6 (2)	C15—C14—H14B	110.5
C2—C3—H3	119.2	C13—C14—H14B	110.5
C4—C3—H3	119.2	H14A—C14—H14B	108.7
N3—C4—C5	127.7 (2)	C14—C15—C11	103.1 (2)
N3—C4—C3	114.9 (2)	C14—C15—H15A	111.1
C5—C4—C3	117.4 (2)	C11—C15—H15A	111.1
N1—C5—C4	119.3 (2)	C14—C15—H15B	111.1
N1—C5—C6	113.3 (2)	C11—C15—H15B	111.1
C4—C5—C6	127.5 (2)	H15A—C15—H15B	109.1
N2—C6—C7	119.5 (2)	O1—C16—Re1	177.8 (3)
N2—C6—C5	114.9 (2)	O2—C17—Re1	177.4 (3)
C7—C6—C5	125.5 (2)	O3—C18—Re1	176.3 (3)

### Hydrogen-bond geometry (Å, º)

**Table:**

*D*—H···*A*	*D*—H	H···*A*	*D*···*A*	*D*—H···*A*
N3—H3*A*···Cl1^i^	0.88	2.53	3.363 (3)	158
N4—H4···Cl1^ii^	0.88	2.65	3.419 (2)	147

Symmetry codes: (i) −*x*+1, −*y*+1, −*z*; (ii) −*x*+3/2, *y*+1/2, *z*.
